# Podoconiosis research to implementation: a call for global action

**DOI:** 10.1016/S2214-109X(18)30298-5

**Published:** 2018-09

**Authors:** Kebede Deribe, Nebiyu Negussu, Mbonigaba Jean Bosco, Gail Davey

**Affiliations:** Wellcome Trust Centre for Global Health Research, Brighton and Sussex Medical School, Brighton, BN1 9PX, UK, School of Public Health, Addis Ababa University, Addis Ababa, Ethiopia; Federal Ministry of Health, Addis Ababa, Ethiopia; Malaria and Other Parasitic Diseases Division, Rwanda Biomedical Center, Kigali, Rwanda; Wellcome Trust Centre for Global Health Research, Brighton and Sussex Medical School, Brighton, BN1 9PX, UK, School of Public Health, Addis Ababa University, Addis Ababa, Ethiopia

Podoconiosis is a form of tropical lymphoedema caused by exposure to volcanic soils in people who do not use footwear.^1^,^2^ The past fifteen years have seen substantial progress in podoconiosis research.^3^,^4^ We are now entering a crucial time marked by the need for increased funding both for research and for translation of this research into implementation. Together, research and its translation will shape a global strategy for podoconiosis control and elimination.^5^,^6^

There have been several encouraging developments in the fight against podoconiosis in recent years. National or local programmes for the control and elimination of podoconiosis have been initiated in four endemic countries (Ethiopia, Uganda, Rwanda, and Cameroon). Nationwide mapping of podoconiosis has been completed in three countries,^6^ and at least three countries have achieved impressive improvements in surveillance systems. Morbidity management services have also been expanded in Ethiopia.^5^

Substantial progress has also been made in terms of research. Genome wide association studies have identified genetic susceptibility loci.^7^ Mineralogical studies have linked specific minerals (smectite, mica, and quartz) within the soil with a high prevalence of podoconiosis.^8^ Social and economic studies have quantified the stigma,^9^ discrimination, and economic impact of the disease.^10^ Developments in geostatistical and machine learning approaches have facilitated disease distribution mapping and estimation of the burden of disease.^11^ Additionally, the effect of a simple lymphoedema management package on the frequency of acute attacks has been documented.^12^

Despite this progress in generating evidence, translation into policy and practice at the global level is poor. Four clear barriers exist. First, at present no global strategy for the control or elimination of podoconiosis exists. Countries are implementing interventions based on experience from pilot projects. Normative guidance about the implementation of podoconiosis interventions with clear end goals must be provided by WHO. Although podoconiosis is mentioned on the WHO website,^1^ the lack of clear global strategies for podoconiosis has significantly limited advocacy and resource mobilisation.

Second, podoconiosis is one of the least financed neglected tropical diseases.^13^ To accelerate progress in the control and elimination of podoconiosis, increased investment is needed. While resources provided by donors and philanthropic organisations are critical, domestic financing of podoconiosis intervention should also be encouraged. Of the 32 countries considered endemic for podoconiosis, 22 are categorised as middle-income countries. These countries could ensure domestic resource allocation for sustainable financing of the prevention and treatment of podoconiosis. The elimination of podoconiosis aligns well with attainment of the UN’s Sustainable Development Goals (SDG).^14^ Podoconiosis interventions are a perfect example of health services requiring universal health coverage (SDG3). Responsive health systems with financial risk protection for affected individuals is critical. Nonetheless, the elimination of podoconiosis depends on the progress of other SDG goals. Determinants and drivers of podoconiosis are directly linked with other goals such as addressing poverty and social protection (SDG1), universal access to safe and affordable water and hygiene (SDG6), working and living conditions (SDG8), and inequalities (SDG10).^14^

Third, there is insufficient innovation in respect to new and better tools for podoconiosis prevention, control, and elimination.^6^ Point-of-care diagnosis, innovations which improve the outcome of morbidity management, and new tools for personal protection from soil exposure, including work-friendly and season-friendly footwear, are all important. To realise the elimination of podoconiosis, innovation that is faster and smarter than the current trend is crucial, as well as using multi-disciplinary approaches to develop tools.

Fourth, reliable data for decision making is essential for effective use of available resources. Investment in distribution mapping and burden estimation in all endemic countries is crucial.^3^ Well-powered surveys will enable estimation of national disease burdens, but countries should also invest in surveillance systems and integrate indicators for podoconiosis into national health data platforms.

The first international conference on podoconiosis will take place on September 23, 2018, in Addis Ababa, Ethiopia. The overall theme of the conference is “Research to implementation: a call for global action”, and the meeting aims to delineate a clear direction for podoconiosis intervention in endemic countries. The following outcomes are expected from the conference; first, the conference will be an important advocacy forum to showcase the key research and implementation advances achieved so far. Policy makers and programme planners will have the opportunity to share experiences. Second, resource mobilisation and domestic financing will be discussed. Third, a written commitment to global podoconiosis implementation will be generated. This will outline the guiding principles for the implementation and financing of podoconiosis programmes.

Elimination of podoconiosis is achievable within one generation. To achieve this goal, resources for translation of the available evidence into practice, for innovation and development of new tools, and for development of a comprehensive global strategy and case for investment are essential.
